# Linking systemic diseases with oral health: Awareness among general physician

**DOI:** 10.6026/973206300210841

**Published:** 2025-04-30

**Authors:** Pranav D Patil, Pradnya S. Jadhav, Sivaponnappan K., Punitha Gnanaselvi U., Kamatchi D., Rajmohan Maruthavanan

**Affiliations:** 1 Department of Conservative Dentistry & Endodontics, Bharati Vidyapeeth Dental College and Hospital, Sangli, Maharashtra, India; 2 Department of Public Health Dentistry, Government Dental College and Hospital, Chhatrapati, Sambhajinagar, Maharashtra, India; 3 Department of Dental Surgery, Govt. Medical College, Sivagangai, Tamilnadu, India; 4 Department of Dental surgery, KAPV Govt medical College, Trichy, Tamilnadu, India

**Keywords:** Oral health, systemic disease, general physician, awareness, interdisciplinary care, healthcare integration

## Abstract

The relationship between oral health and systemic disease exists. However, its understanding among physician is inadequate and less comprehensive in nature. Hence, a survey of 135 general physicians showed that 53.3% possessed moderate awareness and 24.4% had high awareness while 22.2% had low awareness. Knowledge levels of physicians who had more than ten years of experience proved to be significantly better than others (p < 0.05). The practice of asking about oral health occurred regularly for less than 40% of providers while dental referrals reached only 28% of necessary patients. Interdisciplinary education at an elevated level should become mandatory to develop better integrated treatment approaches.

## Background:

General health and well-being strongly depend on maintaining healthy oral health during life. Multiple studies now show that oral conditions create a two-way path toward different systemic diseases including diabetes mellitus as well as cardiovascular disease and respiratory infections [1, 2]. Increased awareness of the oral-systemic disease links was significantly associated with the referral of patients to dentists and belief in improved patients' access to oral care services [3, 4]. General physicians show inconsistent knowledge about the interrelated nature of oral-health to systemic conditions. As the initial healthcare providers in hospitals physicians have an excellent opportunity to notice early oral disease indicators of systemic conditions or arrange dental examinations for their patients when needed [5]. The insufficient dental education about dentistry which medical students receive during their undergraduate training leads to an insufficient level of knowledge and practice among physicians [6]. Several research studies during the recent period demonstrate that systemic conditions affect both oral disease initiation and oral health progression in parallel directions. Individuals with unregulated diabetes face greater risks of periodontal infections since periodontal therapy succeeded in improving their blood glucose management according to research [7]. The harmful microorganisms from oral bacteria cause bacterial endocarditis, a severe heart condition by passing through inflammation of the gingiva during dental interventions [8]. The associations emphasize how physicians need to include oral health evaluation and management within their standard medical practices. General physicians do not include oral examination as part of their systemic disease evaluations although growing evidence demonstrates its significance. Medical students who do not receive proper dental education fail to understand the oral indications of conditions such as leukemia, HIV/AIDS and anemia that often display symptoms like bleeding gums and mouth lesions and mucosal discoloration [9, 10]. The educational deficiency results in late medical diagnosis alongside improper treatment recommendations that slow down patient care delivery and potentially worsens underlying systemic disease. The practice of medical professionals exchanging information with dental specialists remains rare because many healthcare facilities lack well-integrated healthcare models. Medical and dental services operate independently from each other within various healthcare settings which lowers the potential for collaborative work. Inter professional education enhancement combined with structured referral systems serves to improve patient-care interconnectedness by supporting whole-person medical treatment approaches [11]. General physicians enhance patient care when they understand how oral diseases affect entire body systems to help in both preventing issues and detecting conditions early. Improved knowledge about oral-systemic connections by general physicians will lead to better patient results and integrated healthcare practice. Therefore, it is of interest to evaluate the knowledge of general physicians to relate disorders affecting the body system with oral health.

## Materials and Methods:

The researchers conducted their cross-sectional survey for three months across medical practitioners at urban and semi-urban locations. Orthodontists participated from hospitals together with private clinics and community health centers. The study obtained ethical permission from the institutional review board before starting operations. A pre-validated questionnaire measuring the knowledge levels of general physicians concerning systemic disease and oral health connections was specifically formed for the study. The 20-question survey had questions of both demographic nature and knowledge-level questions and clinical practice-oriented inquiries about oral-systemic health. The researchers selected 150 general physicians using convenience sampling. Every participant voluntarily consented to take part in the study before researchers collected information. The survey distribution method included face-to-face encounters and digital transmission through different electronic platforms with the goal of boosting participant engagement. Researchers utilized SPSS version 25 to collect and evaluate the obtained data. The researchers used descriptive statistics which included frequency and percentage to provide results about categorical variables. The Chi-square analysis was employed for determining the relationship between awareness status and clinical experience duration and practice type at p < 0.05 significance.

## Results:

Out of the 150 general physicians approached, 135 responded to the survey, resulting in a response rate of 90%. Among them, 78 (57.8%) were male and 57 (42.2%) were female. The majority of participants (46.7%) had between 5 to 10 years of clinical experience, while 30.4% had more than 10 years, and 22.9% had less than 5 years of practice (Table 1). Regarding awareness levels, 33 physicians (24.4%) had high awareness, 72 (53.3%) demonstrated moderate awareness, and 30 (22.2%) showed low awareness about the link between systemic and oral health (Table 2). A statistically significant association was found between years of experience and awareness level (p = 0.03). When asked about their clinical practices, only 54 participants (40%) reported routinely inquiring about oral health during patient assessments. Furthermore, only 38 physicians (28.1%) referred patients to dental professionals when systemic diseases presented oral manifestations (Table 3). These findings suggest a moderate level of awareness but highlight gaps in practice among general physicians (Table 2 and Table 3).

## Discussion:

This investigation was designed to examine the knowledge level and treatment behavior of regular physicians about interconnections between general medical health and dental wellness. The study results showed participants had moderate awareness because 24.4% demonstrated high awareness but 22.2% displayed low awareness. A number of research studies have confirmed that general medical practitioners show limited knowledge integration regarding oral health care [1, 2]. Medical research shows strong evidence between systemic health conditions such as diabetes mellitus, cardiovascular disease and respiratory illnesses and the presence of periodontitis [3, 4-5]. A considerable portion of physicians from our study did not integrate oral health assessment as part of their regular patient check-ups. Primary care facilities worldwide demonstrate a common pattern of neglecting oral health priority [6, 7]. Results show that physician reference of patients to dental services is inadequate because only 28.1% of participants referred patients who exhibited systemic condition oral manifestations. Researchers from both developed and developing countries have demonstrated that interprofessional relations between dental and medical practitioners maintain insufficient levels of interaction [8, 9-10]. Most dentists were aware of the oral-systemic link. They believed that patients' access to oral care would improve if they were aware of a connection between oral and systemic health [11, 12]. This knowledge deficiency among new practitioners exists since medical curricula typically lack formal oral health instructions [13]. The solution needs complete educational reforms which should include oral health topics throughout medical education for both undergraduate students and post-graduate levels. Knowledge regarding dental care of pregnant women especially as during pregnancy women may acquire new habits relevant to the oral health of their children is mandatory [14]. The study confronts two main issues because it depends on self-reported information together with its cross-sectional research design approach. According to the results there is an urgent need for team-based medical professionals to collaborate and understand the vital aspects of complete patient treatment [15].

## Conclusion:

General physicians show an average comprehension of oral health relations to systemic conditions although their knowledge has considerable gaps as their procedures vary. Medical education needs integration of oral health education together with dental professional collaboration to achieve essential outcomes. Awareness leads patients to seek diagnoses and medical professionals to make timely referrals so as to achieve better healthcare results.

## Figures and Tables

**Table 1:** Demographic distribution of participants (n = 135)

**Variable**	**Frequency (n)**	**Percentage (%)**
Gender		
Male	78	57.8
Female	57	42.2
Years of Practice		
< 5 years	31	22.9
5-10 years	63	46.7
> 10 years	41	30.4

**Table 2:** Awareness level of participants regarding oral-systemic health links

**Awareness Level**	**Frequency (n)**	**Percentage (%)**
Low	30	22.2
Moderate	72	53.3
High	33	24.4

**Table 3:** Clinical practices related to oral health among physicians

**Practice Question**	**Yes (n %)**	**No (n %)**
Do you routinely inquire about oral health?	54 (40%)	81 (60%)
Do you refer patients to dentists when needed?	38 (28.1%)	97 (71.9%)
